# Peritoneal carcinomatosis: limits of diagnosis and the case for liquid biopsy

**DOI:** 10.18632/oncotarget.16480

**Published:** 2017-03-22

**Authors:** James R.W. McMullen, Matthew Selleck, Nathan R. Wall, Maheswari Senthil

**Affiliations:** ^1^ Department of Basic Sciences, Center for Health Disparities & Molecular Medicine, Division of Biochemistry, Loma Linda University Medical Center, Loma Linda, CA, USA; ^2^ Department of Surgery, Division of Surgical Oncology, Loma Linda University Medical Center, Loma Linda, CA, USA

**Keywords:** peritoneal carcinomatosis, liquid biopsy, biomarker, exosomes

## Abstract

Peritoneal Carcinomatosis (PC) is a late stage manifestation of several gastrointestinal malignancies including appendiceal, colorectal, and gastric cancer. In PC, tumors metastasize to and deposit on the peritoneal surface and often leave patients with only palliative treatment options. For colorectal PC, median survival is approximately five months, and palliative systemic therapy is able to extend this to approximately 12 months. However, cytoreductive surgery with hyperthermic intraperitoneal chemotherapy (CRS/HIPEC) with a curative intent is possible in some patients with limited tumor burden. In well-selected patients undergoing complete cytoreduction, median survival has been reported as high as 63 month. Identifying patients earlier who are either at risk for, or who have recently developed PC may provide them with additional treatment options such as CRS/HIPEC. PC is diagnosed late by imaging findings or often times during an invasive procedures such as laparoscopy or laparotomy. In order to improve the outcomes of PC patients, a minimally invasive, accurate, and specific PC screening method needs to be developed. By utilizing circulating PC biomarkers in the serum of patients, a “liquid biopsy,” may be able to be generated to allow a tailored treatment plan and early intervention. Exosomes, stable patient-derived nanovesicles present in blood, urine, and many other bodily fluids, show promise as a tool for the evaluation of labile biomarkers. If liquid biopsies can be perfected in PC, manifestations of this cancer may be more effectively treated, thus offering improved survival.

## INTRODUCTION

Peritoneal Carcinomatosis (PC) is a late stage manifestation of several gastrointestinal malignancies characterized by tumor deposition across the peritoneal surface [1]. This can be entirely asymptomatic in its early stages, or as the disease progresses, symptoms such as nausea, diarrhea, abdominal pain, bloating, and weight loss may develop [1]. The disease is often discovered when ascites or intestinal obstruction develop, occurring generally with greater tumor burden which is more difficult to treat [2]. Early PC detection when there is limited tumor burden may increase the effectiveness of current treatment options [3].

Colorectal cancer (CRC), the third most common cancer in the world, provides a good case study of PC. In 2016 there will be an estimated 95,270 cases of colorectal cancer in the United States and nearly 1.4 million cases worldwide [4]. Synchronous PC is diagnosed around the time of diagnosis of primary tumor while metachronous PC is diagnosed at a later time, typically months to years after the original diagnosis [5]. The incidence of synchronous isolated peritoneal carcinomatosis in patients with CRC varies somewhat in the literature from 4%-18% [6–9]. This may even be a low estimate given the lack of sensitivity of imaging for PC and that it may not be discovered until surgical exploration. Meanwhile, metachronous PC has been reported in 5-19% of patients following definitive treatment.

## CYTOREDUCTIVE SURGERY (CRS)

**Figure 1:**
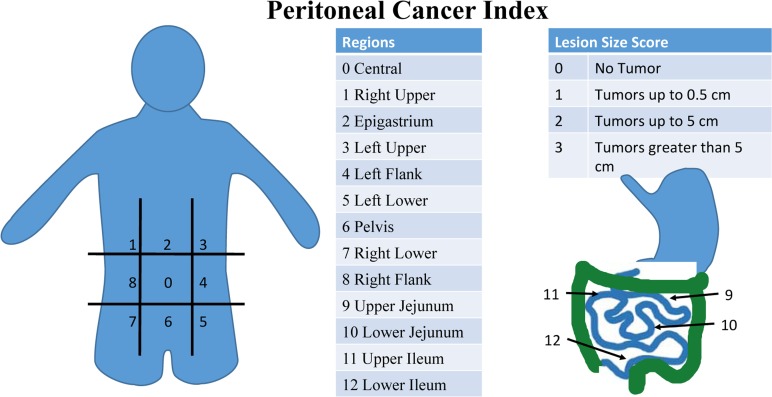
Peritoneal Cancer Index (PCI) scoring system PCI is a diagnostic and prognostic tool that is a sum of scores in thirteen abdominal regions. Each receives a score of 0-3 based on the largest tumor size in each region. Scores range from 0 to 39. Higher scores indicate more widespread and/or larger tumors in the peritoneal cavity.

PC used to be considered a lethal disease with no curative surgical options. However, the growing acceptance of CRS with hyperthermic intraperitoneal chemotherapy (HIPEC), has offered the possibility of improved survival for carefully selected patients [3]. This method makes use of “cytoreduction” to surgically remove gross visible tumor deposits followed by direct contact of heated cytotoxic chemotherapy agents to affect any residual disease. Given during surgery, this protocol maximizes potential contact with the peritoneal surface while minimizing systemic toxicity. Specifically, hyperthermia between 41 and 43 degrees centigrade is combined with large molecular weight drugs that penetrate between a few cells deep to 3mm causing cytotoxic effects [10]. Dr. Paul Sugarbaker is credited with developing this treatment option by combing these elements into a curative approach to peritoneal dissemination of gastrointestinal malignancies [11, 12]. Median survival of CRC PC without any treatment is approximately 4-7 months, while palliative systemic therapy may extend this to 12-23 months based on several series [13–15]. Median overall survival with CRS/HIPEC has been reported to range from 22 to 63 months with a 5-year survival of 40-51% in selected patients [13, 15, 16]. The outcomes of CRS/HIPEC are strongly influenced by careful patient selection and complete cytoreduction (CC-0) (see Table 1) [17]. Survival of patients with colorectal cancer who receive less than complete cytoreduction (CC-1 or CC-2) or have a higher burden of disease as indicated by the peritoneal carcinomatosis index (PCI) (see Figure 1) is significantly diminished compared to that of a CC-0 resection [17–19]. Extensive disease burden at identification often leaves patients with only palliative treatment options [20]. Despite the benefit of CRS/HIPEC, only about 25% of patients with PC will be eligible for this approach given the late presentation and burden of disease. In order to expand patient eligibility and offer treatment with a curative intent, early detection of PC, before significant tumor burden develops, is essential.

**Table 1:** CC is the completeness of cytoreduction score

Completeness of Cytoreduction scores	
Score	Size of largest post-surgery residual tumor
CC-0	No visible tumor
CC-1	Less than 0.25 cm
CC-2	Between 0.25 cm and 2.5 cm
CC-3	> 2.5 cm or confluent

## IMAGING

Traditional imaging, such as CT and MRI lack sensitivity to both detect and estimate disease burden in PC. Classic computed tomography (CT) signs of PC such as “omental caking,” thickening of the omentum, and peritoneal nodules are not common radiographic findings in “early” disease states. Several studies designed to determine the specificity and accuracy of CT scans in assessing tumor burden for PC found that CT significantly underestimated the amount of disease present in the peritoneal cavity [21–24]. Sensitivity for CT detection of tumor nodules less than 0.5 cm and 1cm had been reported to be 11% and 25-50% respectively [23]. This is particularly important in colorectal PC, where PCI is a critical determinant of complete cytoreduction and long-term outcomes [25]. In a study by Koh et al looking specifically at CRC PC they determined CT significantly underestimated clinical PCI [23]. In fact sensitivity of small bowel involvement in each region ranged from 8-17%. Despite this, the Fifth International Workshop on Peritoneal Surface Malignancy in Milan, identified CT as the principal imaging modality to assess suitability for CRS [26]. Ultrasound also has very limited sensitivity for detecting PC nodules [27–29]. Magnetic resonance imaging (MRI), and particularly diffusion weighted images, has been demonstrated in prospective studies to have increased accuracy in detection of carcinomatosis within certain areas of the abdomen [30]. This however carries its own limitations due to the motion artifacts of peristalsis, cost, and the need for radiologists trained in their interpretation and inter-observer variation. Additionally, positron emission technology (PET) may have increased sensitivity, but similar limitations and absence of added clinical value often precludes its use for determining resectability [24, 31–33]. These difficulties, especially limited sensitivity, lack of meaningful clinical correlation and high cost, reduce the utility of non-invasive imaging in the early detection of PC. With the current technology, laparoscopy or exploratory surgery is often necessary to confirm the diagnosis and extent of PC (see Table 2) [34].

**Table 2:** This table summarizes the pros and cons of each non-invasive imaging modality in assessing PC

Non-Invasive Imaging Utility in PC Detection				
Image Modality	Pros	Cons	Sensitivity/Specificity as compared to surgical analysis	References
Ultrasound	Inexpensive, Effective for ascites detection	Limited PC nodule sensitivity, highly operator dependent	Non-specific [26]	[24, 25]
CT	Standard staging workup	Limited small PC nodule sensitivity, Inter-observer variability	25-100%/78-100% with only 11-48% sensitivity for tumors less than 5 mm [26]	[18–21]
MRI	High PC sensitivity	Relatively Expensive, slight peristalsis motion artifact, inter-observer variability	90%/95.5% (diffusion weighted) [26]	[28]
PET/ PET-CT	High PC sensitivity	Relatively Expensive, Peristalsis motion artifact	78-97%/55-90% [26]	[27, 28]

## LIQUID BIOPSY

Given the narrow subset of patients who are offered CRS/HIPEC, a potentially life-saving treatment, we are in need of a paradigm change. Patients who are either at risk for developing PC or who are in the earliest stage of this disease process may significantly benefit from expanded treatment options. The term “liquid biopsy” has reached prolific use as large-scale investigations seek to identify tumor markers in the serum. This usually refers to molecular diagnostic studies that are performed on blood or body fluid as opposed to cancerous tissue itself [35]. Multiple serum tumor markers: carcinoembryonic antigen (CEA), carbohydrate antigen CA 19-9, and CA 125, are commonly elevated in patients with PC and the degree of elevation tends to correlate with the extent of PC [36]. However, these serum tumor markers are inadequate for early detection of PC. Moreover, they lack specificity to predict the presence or risk of PC in patients with CRC. There is a critical clinical need to identify circulating tumor biomarkers of aggressiveness, likelihood of recurrence, risk of metastasis such as PC, or even the presence of a malignancy to better tailor therapy for patients. For example, if a patient with a newly diagnosed stage III colorectal cancer is known to be at significant risk for peritoneal recurrence due to the presence of a specific set of biomarkers in their serum, they may benefit from prophylactic HIPEC. This is just one example of how this technology may be applied.

If blood-borne biomarkers for PC with high sensitivity and specificity are discovered, patients developing PC may be quickly identified with a blood test, a liquid biopsy. One such type of biomarker, microRNAs (miRs), short, non-coding RNAs that regulate mRNAs, has demonstrated diagnostic utility by correctly identifying several cancers of unknown primary with reasonable accuracy [37]. The diagnostic miR profile that was used in this study was generated from miR analysis in well differentiated primary tumors [37]. Several miRs, such as miR-21, have been linked to gastrointestinal cancers as potential diagnostic targets and prognostic indicators [38]. However these miRs and other types of RNAs are rapidly degraded in the plasma [39–41].

## EXOSOMES

**Figure 2:**
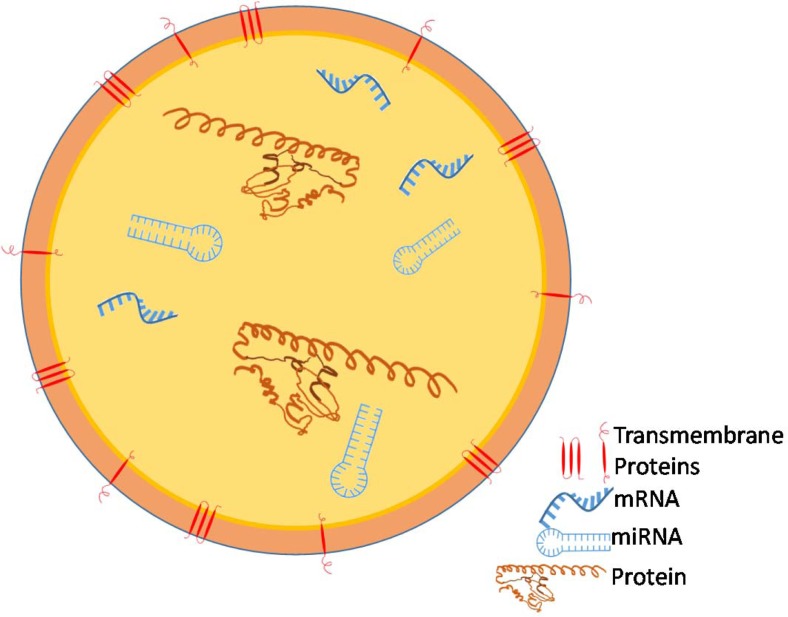
Tumor Cells release nanovesicles called exosomes which carry RNAs, including microRNAs and messenger RNAs, and proteins

Exosomes, small cell-derived vesicles (Figure 2), can protect RNAs and miRNAs, from being degraded [42–46]. When researchers exposed miRs to RNase, the miRs that were in exosomes and cells were protected while the free RNAs were degraded [42]. When exosomes were exposed to RNase the contained RNAs were protected from degradation while cellular RNA was degraded by the same RNase [45]. Exosomes hold great potential for both diagnosis and prognosis of diseases and are exceptionally useful as cancer biomarkers [47]. When a panel of lung cancer associated miRs was examined in solid tumors and tumor exosomes from patient plasma, most of the miRs were found to have highly comparable expression levels (see Table 3 for the miRs) [48, 49]. Cervical cancer cell line tumor derived exosomes (TEXs) contain survivin, which contributes to cancer aggressiveness and metastatic potential [50, 51]. In a study of ovarian cancer, greater numbers of cancer exosomes were found in the serum as more advanced cancer stages were examined [52]. Furthermore, approximately four times more serum exosomes were discovered in lung cancer patients as compared to cancer free controls and the exosomes contained more than twice the miRs [48]. In our research, we found increased serum exosome levels in patients with prostate and breast cancers as compared to disease free controls [53–55]. TEXs are prevalent in patient serum from multiple cancer types and protect labile biomarkers from degradation [42, 45, 48, 53–55].

**Table 3:** These lung cancer associated miRs were discovered in both solid tumor and in tumor exosomes

miR-17-3p	miR-21	miR-106a	miR-146	miR-155	miR-191
miR-192	miR-203	miR-205	miR-210	miR-212	miR-214

Exosomes have not been extensively studied in PC diagnostics. Andre and colleagues examined ascites exosomes from patients with PC and found that the tumor specific markers Her2/Neu, TRP1, and Mart1 were present in ascites tumor exosomes [56]. Tokuhisa and colleagues have identified several RNAs present in exosomes within peritoneal ascites, peritoneal lavage, and PC metastatic cell lines [57]. After exosome miR screening, 5 miRNAs were selected as a panel of significantly differentially regulated RNAs: miR 1202, 1207-5p, 1225-5p, 320c, and 4270 [57]. miR 21 had the strongest signal intensity in malignant ascites [57]. Exosomes from peritoneal lavage were probed for miR 21, 1225-5p, and 320c; miR 21 and 1225-5p were found to be upregulated in later stages of gastric cancer and correlated with serosal invasion [57]. In a study on primary gastric cancer tissue, miR 1255-5p was generally downregulated and was found to inhibit cancer cell growth, motility, as well as cancer invasion [58]. This apparently contradictory result suggests that miR 1225-5p in peritoneal lavage either is being released by non-cancerous tissue in an attempt to stop abnormal growths or is being used by cancers in order to facilitate better attachment to the peritoneum. In apparently contradictory results to the above study, in gastric cancer cell lines and *in vivo*, miR 1255-5p was generally downregulated and was found to inhibit cell growth and motility as well as cancer invasion [58].

The prospective gastric PC miR biomarkers described in the study by Tokuhisa and colleagues [57] have been found to be associated with other cancers as well (see Table 4). Notably, miR 1202, 1207-5p, 1225-5p, and 4270, were found circulating in the blood of breast cancer patients [59]. In human bladder cancer tissue samples, miR 320c was significantly downregulated [60]. In hepatocellular carcinoma tissue samples, miR 1207-5p was found to be significantly downregulated [61]. In adrenocortical carcinoma tissue samples, increased miR 1202 expression was found to be associated with significantly reduced patient lifespan [62]. The gaps in our knowledge of RNA signaling in cancer are immediately apparent from Table 4. The observation that these miRs, either individually or in a group, are associated with multiple types of cancer and are found in exosomes from the peritoneal cavity suggest the potential of exosomal diagnosis of PC.

**Table 4:** Gastric Cancer PC associated exosomal miRs and their prevalence in various cancers

	Gastric Cancer	Lung Cancer	Liver Cancer	Breast Cancer	Prostate Cancer	Colorectal Cancer	Adrenal Cancer
miR 1202	↔malignant ascites, peritoneal lavage fluid, cell culture [52]	X	X	↑ serum exosomes [54]	X	X	↑Ø tumor tissue [62]
miR 1207-5p	↔malignant ascites, peritoneal lavage fluid, cell culture [52], ↓tNm tumor tissue [63], ↓ tumor tissue [64]	X	↓ solid tumor [56]	↑ serum exosomes [54]	↑ serum [65]	↑ solid tumor [66]	X
miR 1225-5p	↑tnM peritoneal lavage fluid [52], ↓ cancer tissue [53]	X	↓ blood [67]	↑ serum exosomes [54]	↑ blood [68], ↓ solid tumor [69]	↓ solid tumor [70]	↓ multiple types of solid tumors [71]
miR 320c	↔malignant ascites, peritoneal lavage fluid, cell culture [52]	↓ solid tumor [72]	X	X	X	X	X
miR 4270	↔malignant ascites, peritoneal lavage fluid, cell culture [52]	X	X	↑ serum exosomes [54]	X	X	X

All data is from human studies

↑, ↓-increased/decreased expression associated with cancer

↑tnM, ↓tnM -increased/decreased expression associated with metastatic cancer

↑tNm, ↓tNm -increased/decreased expression associated with lymph node metastasis

↑ Ø, ↓Ø -increased/decreased expression associated with shorter patient survival

↔-present but no significant differential regulation

X -no research discovered in literature

## RNAS AND GASTRIC CANCER

RNAs are found to be globally downregulated in cancer [37]. Upregulated RNAs and miRs are likely to be related to cancer growth and function or the body's response to cancer. miR 320c inhibited cell growth and motility in bladder cancer [60]. miR 1207-5p functioned to inhibit cell growth and invasion in liver cancer but functions to increase stemness in colorectal cancer [61, 63]. Since these exosomal-associated miRs have known functions in tumors, the possibility emerges of tailoring PC treatment based on what biomarkers are discovered in a patient's exosomes. miR 320c inhibited cell growth and motility and miR 1207-5p functioned to inhibit cell growth and invasion. The observation that these exosomal-associated miRs have known functions in tumors opens up the possibility of utilizing these biomarkers to tailor an individual patient's treatment. [60, 61]

## FUTURE DIRECTIONS

Additional research is needed to identify biomarkers in peritoneal carcinomatosis. Distinguishing metastatic disease of peritoneal origin from solid organ metastases should be both biologically feasible and clinically useful. As we look to exosomes to provide insight into the field, shared challenges in nanovesicle isolation and validation will need to be addressed. Reliable and efficient methods as well as recognized standards will need to be established for clinical use. Future clinical work in this field should include the prospective collection of samples for retrospective investigation. This will be instrumental in establishing clinical validity and utility. We are hopeful that the shared work of many will continue to yield advances in reducing the burden of life lost from this aggressive malady.

## CONCLUSIONS

Recently, TEXs have been implicated in facilitating metastasis. TEXs can be taken up by multiple cell types, including endothelial cells, bone marrow progenitor cells, and other cancer cells [64–67]. These exosomes demonstrated the ability to deliver functional RNAs and proteins to recipient cells, modifying their growth patterns to be pro-oncogenic [65, 66]. Further, TEXs have demonstrated the ability to greatly increase metastatic tumor burden in a mouse model [64]. Studying TEXs in the context of metastasis is a promising field. PC is currently difficult to detect at its onset. Late PC detection usually leaves the disease incurable. PC must be detected sooner for better patient outcomes. Non-invasive imaging is impractical for early PC detection. Detection of PC by means of markers within a patient's biofluids, such as a serum “liquid biopsy” would be ideal. Both serum and ascites contain biomarkers released by PC. Exosomes are released into serum by multiple types of cancers and protect their contents from degradation in the blood. These provide us with a likely source of a sensitive and specific diagnostic modality to detect PC in its earliest most treatable from.
